# *Rap2A* Is Upregulated in Invasive Cells Dissected from Follicular Thyroid Cancer

**DOI:** 10.4061/2011/979840

**Published:** 2011-10-20

**Authors:** Indira Prabakaran, Jillian R. Grau, Robert Lewis, Douglas L. Fraker, Marina A. Guvakova

**Affiliations:** ^1^Department of Surgery, School of Medicine, University of Pennsylvania, Philadelphia, PA 19104, USA; ^2^Department of Pathology and Laboratory Medicine, School of Medicine, University of Pennsylvania, Philadelphia, PA 19104, USA

## Abstract

The development of molecular biomarkers (BMs) of follicular thyroid carcinoma is aimed at advancing diagnosis of follicular neoplasm, as histological examination of those tumors does not lend itself to definitive diagnosis of carcinoma. We assessed the relative levels of expression of 6 genes: *CCND2, PCSK2, PLAB, RAP2A, TSHR, and IGF-1R* in archived thyroid tissue. The quantitative real-time PCR analysis revealed a significant change in 3 genes: *PSCK2* (a 22.4-fold decrease, *P* = 2.81*E* − 2), *PLAB* (an 8.3-fold increase, *P* = 9.81*E* − 12), and *RAP2A* (a 6.3-fold increase, *P* = 9.13*E* − 10) in carcinoma compared with adenoma. Expression of *PCSK2* was equally low, *PLAB* was equally high, whereas *RAP2A* expression was significantly higher (25.9-fold, *P* = 0.039) in microdissected carcinoma cells that have invaded through the thyroid capsule and entered blood vessels than in thyroid tumor cells growing under the capsule. Thus, *RAP2A* appeared as a unique and worthy of further evaluation candidate BM associated with invasion of thyroid follicular cells.

## 1. Introduction

Differentiated thyroid carcinomas originating from the follicular epithelium have a papillary (range, 65–88%) and a follicular (range, 9–23%) histotype [1]. Although follicular thyroid carcinomas (FTCs) are the second most common differentiated thyroid cancers, they are more aggressive than papillary thyroid carcinomas (PTCs) and invade into the capsule (minimally invasive) and veins (angioinvasive) within the thyroid gland. Importantly, mortality is related to the degree of invasion [2]. Furthermore, FTC has a greater rate of recurrence and is frequently associated with distant metastasis to the lung, bone, brain, and liver [3, 4]. Total thyroidectomy represents the dominant method of surgical treatment for follicular neoplasms diagnosed preoperatively by fine needle aspirates (FNAs). Distinguishing follicular adenoma from minimally invasive or encapsulated angioinvasive carcinoma in FNA can be extremely challenging [3, 5]. Gene and micro-RNA (miRNA) expression profiling are being investigated to identify potential BMs differentiating benign from malignant follicular tumors [6, 7]. Such BMs might be clinically useful to help predicting follicular thyroid malignancy and reduce the frequency of surgical procedures by identifying those patients with benign lesions who do not require surgical excision. So far, however, global genetic screens have not improved preoperative diagnosis of FTC. Hence, novel approaches are necessary to identify potential preoperative molecular BMs to facilitate the diagnosis of FTC. One of the approaches could be discovering specific molecular BMs associated with invasion of thyroid follicular cells.

## 2. Materials and Methods

### 2.1. Thyroid Tissue

Cases of follicular-patterned thyroid cancer are quite rare; even lesser is the number of remaining samples available for research. For this study, a unique cohort of patients diagnosed with follicular-patterned thyroid cancer was identified on review of medical records from the Hospital of University of Pennsylvania between 1992 and 2007. After reexamination of 16 available formalin-fixed, paraffin-embedded (FFPE) tissues (for histological presence of vascular and/or capsular invasion) and initial determination of integrity of total RNA in the tissue scrapes, we found that two samples had degraded RNA, one sample had too little RNA to be amplified by *in vitro* transcription (IVT), in two samples the areas of invasion had already been cut through, and 10 specimens fully met study's criteria. Subsequently, the study was performed in specimens from 8 patients diagnosed with FTC, 1 patient diagnosed with FTC-Hürthle cell carcinoma (HCC), 1 patient diagnosed with HCC, and 10 patients diagnosed with follicular thyroid adenoma (FTA). Groups of patients with FTA (mean age, 52.4 ± 16.2 SD years) and follicular thyroid malignancy (mean age, 50.8 ± 13.1 SD, years) were age matched (Table 1). Ten normal FFPE thyroid samples were from patients who underwent surgery after diagnosis of larynx squamous cell carcinoma (mean age, 62.4 ± 7.0 SD, years). Histopathological analysis of all tissues was performed by a surgical pathology fellow (JG) and confirmed by a thyroid pathologist (Dr. Virginia LiVolsi). The study protocol was approved by the University of Pennsylvania Institutional Review Board committee.

### 2.2. Thyroid Tissue Analysis: RNA Extraction, cDNA Synthesis, and Quantitative Real-Time PCR (Q-RT-PCR)

RNA was extracted from the normal, adenoma, and cancer tissue scrapes using the Absolutely RNA FFPE kit (Stratagene, La Jolla, CA). In addition, RNA was extracted from a snap frozen thyroid carcinoma using the High Pure RNA Tissue kit (Roche Diagnostics, Indianapolis, IN) to use as a positive control and generate a standard curve for all subsequent PCR reactions. Integrity of RNA from a snap frozen tissue was determined by 260 to 280 nm ratio using a DU 640 spectrophotometer (Beckman Coulter, Fullerton, CA). Integrity of the scraped tissue RNA was assessed by Q-RT-PCR using 3′*ACTB* and 5′*ACTB* primers (Table 2) and the Paradise Sample Quality Assessment Kit (Molecular Devices, Sunnyvale, CA). 10–100 ng of the scraped tissue RNA or 500 ng of a positive control RNA were reverse-transcribed into single-stranded cDNA using the first-strand cDNA synthesis kit (Roche Diagnostics, Indianapolis, IN). cDNA synthesis was carried out in a 20 *μ*L reaction mix containing 5 mM MgCl_2_, 1 mM dNTPs, 0.04 units of random primers p(dN)_6_, 50 units of RNase inhibitor, and 20 units of Avian Myeloblastosis Virus (AMV) reverse transcriptase. Q-RT-PCR was performed using 3 *μ*L of the first-strand cDNA with 1 *μ*M of the housekeeping gene, *ACTB*, or target gene-specific primers (Table 2) using the LightCycler 2.0 (Roche Molecular Biochemicals, Mannheim, Germany) instrument and the LightCycler Fast Start DNA Master^PLUS^ SYBR Green 1 kit (Roche Diagnostics, Indianapolis, IN) according to the manufacturer's instructions. PCR parameters were a 10 min preincubation time at 95°C followed by 45 cycles of denaturation (10 sec at 95°C), annealing (10 sec at 55°C), and extension (25 sec at 72°C). A standard curve for each of the target and housekeeping gene was generated for every PCR run to determine levels of gene expression. All reactions were performed in duplicates with at least three repeats. Relative expression of each target gene in all samples was determined as a ratio of mRNA of target gene to mRNA of the housekeeping gene as described in [8].

### 2.3. Laser-Capture Microdissection (LCM)

LCM was performed as in the frozen thyroid tissue samples [9] with modifications. Briefly, FFPE blocks of FTC were cut into 7 *μ*m thick sections, mounted on RNase-free membrane slides (MMI, Manchester, NH), deparaffinated with d-limonene, rehydrated with sequential washes of 100%, 95%, and 75% ethanol, and then washed in nuclease-free water. Next, slides were stained with Paradise staining solution (Arcturus Engineering Inc., Mountain View, CA), dehydrated in Xylene for a minimum of 5 min, and air dried. Cells from areas of angioinvasion, capsular invasion, and tumor under the capsule were dissected onto Capsure HS LCM Caps (MMI, Manchester, NH) using a Laser Capture Micro-dissection microscope Nikon ECLIPSE TE 2000-S and MMI Cell Tools software (MMI, Manchester, NH).

### 2.4. Dissected Thyroid Cancer Cell Analysis: RNA Extraction and Amplification, cDNA Synthesis, and Q-RT-PCR

RNA was extracted from laser-captured microdissected cancer cells using the Absolutely RNA FFPE kit (Stratagene, La Jolla, CA). Assessment of the integrity of cellular RNA was performed by Q-RT-PCR using 3′*ACTB* and 5′*ACTB* primers. Amplification of RNA from laser-captured microdissected cells was performed using the Ambion MessageAmp II aRNA kit (Ambion, Austin, TX). We used the IVT method which is based on the linear amplification protocol developed and validated previously [10, 11]. The advantage of such a technique is that the product of the reaction is unable to act as template and the yield of any individual species within a mixed population is for the most part determined by the template concentration that is not changed. Amplification was linear when at least 1ng of LCM RNA was used as the input for IVT. Two rounds of linear amplification of the mRNA fraction of at least 1 ng total cellular RNA were performed. First-strand cDNA synthesis yielded cDNA incorporating a T-7 promoter sequence. This cDNA was converted to a double-stranded transcription template by a second-strand synthesis reaction utilizing exogenous primers that yielded double-stranded cDNA. Double-stranded cDNA was then used as a template for IVT with T7 RNA polymerase to generate amplified antisense RNA (aRNA). Integrity of aRNA samples was determined as described above. aRNA samples with a 3′*ACTB* to 5′*ACTB* ratio of ≤20 or a 260 to 280 nm ratio between 1.8 and 2.2 were used for further experiments. 10–100 ng of aRNA was converted to cDNA using 1 *μ*M target gene-specific primers and the first-strand cDNA synthesis kit (Roche Diagnostics, Indianapolis, IN). Q-RT-PCR was then performed for the housekeeping gene, *ACTB*, and target genes as described above. After all the reactions were performed in duplicates with at least three repeats, relative expression of target genes was determined.

### 2.5. Statistical Analysis

Data were reported as mean ± standard error of the mean (SEM). Comparisons between normal, benign, and cancer groups were made by using one-way analysis of variance (ANOVA). A value of *P* < .05 was considered as statistically significant.

## 3. Results and Discussion

**Figure 1:**
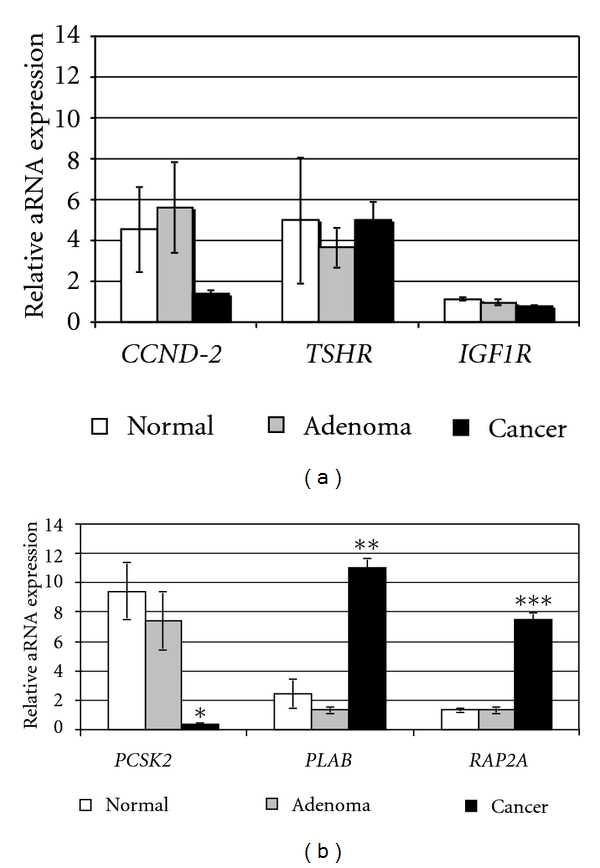
Evaluation of *CCND2, TSHR, IGF-1R* (a) and *PCSK2, PLAB, RAP2A* (b) mRNA expression by Q-RT-PCR in tissue samples from the human thyroid (normal, benign, cancer). All PCR reactions were performed in duplicates with at least three repeats. Mean of normalized expression level of mRNA in each analyzed group (*n* = 10) are shown. Bar, SEM. *P = 2.81*E* − 2, **P = 9.81*E* − 12, and ***P = 9.13*E* − 10.

In this exploratory study, we investigated the expression of the potential thyroid cancer-discriminating genes: *CCND2, PCSK2, PLAB, RAP2A, TSHR*, and* IGF-1R* (Table 2) by comparing their expression at the mRNA levels in the normal thyroid tissue, benign follicular lesions, and follicular carcinomas. The target genes have been chosen based on importance of abnormal expression and activities of the thyroid-stimulating hormone receptor (TSHR) and insulin- like growth factor type I receptor (IGF-IR) in thyroid tumorigenesis [12, 13] and the results of gene micro-array analysis showing differential expression of *CCND2, PCSK2, PLAB, RAP2A* in FTC [14, 15]. We found no statistically significant difference in *CCND2, TSHR*, and *IGF-1R* mRNA expression between the groups of normal thyroid, benign and malignant thyroid cancer (Figure 1(a)). There was no difference between the levels of mRNA expression of *PCSK2, PLAB*, and *RAP2A* between normal thyroid and FTA (Figure 1(b)). Interestingly, however, in the Q-RT-PCR analysis of FTA and cancer, *PSCK2* was markedly downregulated (22.4-fold), whereas *PLAB* and *RAP2A* were notably upregulated (8.3- and 6.3-fold, resp.) in cancer. Furthermore, a comparative mRNA expression analysis revealed a statistically significant difference in *PCSK2* (*P* = 2.81*E* − 2), *PLAB* (*P* = 9.81*E* − 12) and *RAP2A *(*P* = 9.13*E* − 10) expression between groups of benign and malignant thyroid tumors (Figure 1(b)). Thus, in tested age-matched cohort of 20 patients diagnosed with follicular-patterned thyroid neoplasm, the levels of *CCND2, TSHR*, and *IGF-1R* were not significantly different; *PLAB* and *RAP2A* were significantly increased, whereas *PCSK2* was significantly decreased in cancer compared with adenoma. Overexpression of *PLAB *and *PCSK2* as well as down-regulation of* CCND2* has been found in frozen sections of FTC [15]. Weber et al. proposed that a combination of those three genes allowed the accurate molecular classification of FTC versus FTA with a high specificity and sensitivity. However, Shibru et al. were unable to confirm the diagnostic accuracy of the 3-gene assay either in frozen tissue or in FNAs [16]. The difference is likely attributed to the difference in types of analyzed tissue, as Shibru et al. compared a benign group represented by hyperplastic nodule, FTA, Hürthle cell adenomas (HCAs) with a collective group of thyroid malignancies, including FTC, PTC, follicular variant of PTC, HCC. Although FTC and HCC may carry similar molecular alterations [17], PTC has distinct genetic features (somatic alterations such as *RET/PTC* translocation and *BRAF* mutations) that distinguish them from FTC [6]. Our data are in close agreement with the findings reported by Weber et al. except that the observed down-regulation of *CCND2* in cancer has not reached statistical significance.

**Figure 2:**
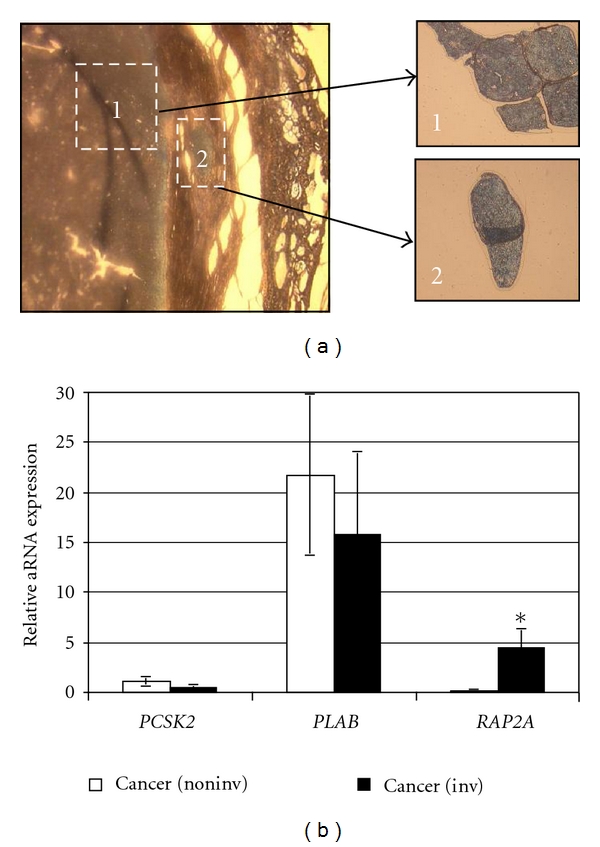
An example of laser-capture micro-dissection in follicular thyroid carcinoma. (a) FTC tissue before (left) and after collecting the groups of cells from the main tumor mass (inset 1) and angioinvasive area (inset 2). Original magnification, 200x. (b) Histogram, relative expression of *PCSK2, PLAB, RAP2A* in the cells collected from matched noninvasive and invasive areas of the same specimens (*n* = 4). Mean of normalized expression levels of aRNA in each group is shown. Bar, SEM. *P = 0.039.

Intratumoral heterogeneity is well-recognized phenomenon [5, 18, 19], so it is plausible that within areas of invasion tumor cells are genetically different from the rest of tumor. Here, we tested the hypothesis that in thyroid malignancy differential expression of molecular BMs may be detected in thyroid follicular cancer cells invaded through the tumor capsule and entered into vasculature. The three genes (*PCSK2, PLAB*, and *RAP2A*) were selected for in-depth analysis because of their significantly different expression in cancers compared with adenomas (Figure 1(b)). To ensure the presence of invasion in freshly cut 7 *μ*m thick sections of cancer tissue, a thyroid pathologist reviewed slides stained with hematoxylin and eosin and marked the areas of invasion using diagnostic criteria adopted in our institution [3]. Nine angioinvasive samples of thyroid cancers had both capsular and vascular invasion; one minimally invasive specimen had only capsular invasion. Eight out of ten specimens had more than one invasive focus. To selectively isolate population of thyroid carcinoma cells that have invaded the capsule to enter blood vessels and to compare them to the cells remained in the main tumor mass, we applied an LCM method as illustrated in Figure 2(a). After dissecting multiple areas, the captured cells from each of the cancer specimens were pooled in to two matched groups: (i) remained under the tumor capsule (“noninvasive” group) and (ii) invaded the capsule and/or entered blood vessels (“invasive” group). The total RNA was assessed in the captured tumor cells, and samples with an adequate amount of input RNA (>1.0 ng) were subjected to two rounds of linear amplification by *in vitro* transcription to further increase the amount of RNA. Twice-amplified aRNA of high quality only was used for a cDNA preparation and Q-RT-PCR with specific primers for target genes. As expected from the analysis of tissue scrapes, *PSCK2 *expression was low in the cells from the main tumor mass; it was insignificantly different (*P* = 0.322) in invasive cells dissected from the same specimens (Figure 2(b)). Likewise, *PLAB* expression was equally high in both types of dissected cells (*P* = 0.698). The results of the Q-RT-PCR analysis for *RAP2A *aRNA were intriguing, as the relative level of *RAP2A *expression was 25.9-fold higher (*P* = 0.039) in the cells dissected from areas of invasion. *RAP2A *encodes Ras-related protein 2a (Rap-2a), a member of the Ras family of small GTPases (Rap1a/b and Rap2a/b/c) that has been reported to induce cytoskeleton rearrangements promoting cell rounding and cell migration [20, 21]. Although activating mutations of Rap have not been reported, up regulation of Rap activating guanine nucleotide exchange factors [22, 23] and down regulation of Rap GTPase-activating proteins promoting Rap inactivation [24, 25] have been found in human tumors including thyroid carcinomas [26]. High levels of expression of Rap2, but not Rap1, have been detected in human thyroid cancer cell lines. Importantly, Rap2 protein expression was several fold higher in anaplastic than in well-differentiated papillary thyroid cancer cells [27]. Furthermore, increased Rap activity has been shown to promote carcinoma cells invasion *in vitro* and *in vivo* [28, 29]. We found up regulation of human gene encoding Rap-2a in follicular thyroid cancer tissue, particularly in the regions enriched with invasive cancer cells. It could be speculated that thyroid tumor cells “require” the genetic changes in *RAP2A*, in addition to* PSCK2 *and *PLAB*, to allow them to invade and/or “maintain and flourish” in the nonnative areas of the tumor capsule and blood vessels. 

## 4. Conclusions

We demonstrated the feasibility of combining LCM and Q-RT-PCR for analysis of gene expression in microscopic clusters dissected from FFPE thyroid tissue. Our study is a first and important step in the assessment of novel molecular BMs associated with invasion of follicular thyroid carcinoma cells, despite the relatively small sample size. Validation of diagnostic applicability of *RAP2A* requires a follow-up work in larger tissue sample sets.

## Figures and Tables

**Table 1:** Clinical data of patients from whom follicular thyroid tumor tissue samples were collected.

Gender	Age, years	FNA diagnosis	Nodule size, cm	Final diagnosis	Invasion vascular/capsular
F	48	Follicular neoplasm	5.6 × 4.0 × 2.4	FTC	+/+
M	64	NA	NA	FTC	+/+
F	51	Follicular neoplasm	2.0 × 1.5 × 1.0	FTC	+/+
F	48	Follicular neoplasm	3.6 × 3.0 × 2.2	FTC	+/+
F	56	NA	NA	FTC	+/+
M	25	NA	NA	FTC	+/+
F	40	Follicular neoplasm	3.0 × 2.5 × 1.5	FTC	+/+
F	59	Follicular neoplasm	3.8 ×1.8 × 1.7	HCC, angioinvasive	+/+
M	45	Follicular neoplasm	3.8 × 3.1 × 2.5	FTC	+/+
M	72	Benign goiter	7.0 × 5.3 × 4.3	FTC and HCC	−/+

M	45	Follicular neoplasm	3.2 × 3.0 × 3.0	FTA	−/−
M	76	Follicular neoplasm	3.1 × 5.3 × 2.6	FTA	−/−
M	41	Follicular neoplasm	3.1 × 2.1 × 1.7	FTA	−/−
F	76	Follicular neoplasm	3.1 × 2.0 × 1.5	FTA	−/−
M	64	Follicular neoplasm	4.5 × 4.4 × 3.3	FTA	−/−
M	60	NA	3.8 × 3.0 × 5.0	FTA	−/−
F	28	Follicular neoplasm	2.5 × 2.2 × 2.0	FTA	−/−
F	52	Follicular neoplasm	5.0 × 4.0 × 3.5	FTA	−/−
F	44	Follicular neoplasm	4.5 × 3.5 × 2.9	FTA	−/−
F	38	Follicular neoplasm	3.1 × 1.9 × 1.5	FTA	−/−

N/A: records were not available.

**Table 2:** A list of tested genes and encoded by them proteins, including the gene and protein accession numbers and corresponding intron-spanning primers used for Q-RT-PCR.

Gene	GenBank	Primer sequence	Protein	Swiss-Prot
*CCND-2*	AY888219	S_CAC TTG TGA TGC CCT GAC TG	G1/S-specific cyclin-D2	P30279
		AS_ACG GTA CTG CTG CAG GCT AT		

*PCSK2 *	BC040546	S_AGC ATA CAA CTC CAA GGT TGC	Proprotein convertase subtilisin/kexin type 2	Q8IWA8
		AS_GCT GTA GAT GTC AAT CAG CTG TG		

*PLAB*	BC008962	S_CAA CCA GAG CTG GGA AGA TT	Placental bone morphogenetic protein	Q99988
		AS_AGA GAT ACG CAG GTG CAG GT		

*RAP2A*	NM 021033	S_AGA TCA TCC GCG TGA AGC	Ras-related protein-2a	P10114
		AS_CCC CAC TCT TCA GCA AGG		

*TSHR*	BC024205	S_GGA TAT GCT TTC AAT GGG ACA	Thyroid-stimulating hormone receptor	P16473
		AS_GCA TCT TTG TCA ATA ACT GTC AGG		

*IGF1R*	NM000875	S_GTG AAA GTG ACG TCC TGC ATT TC	Insulin-like growth factor I receptor	P08069
		AS_CCT TGT AGT AAA CGG TGA AGC TGA		

*3′ACTB*	NP001092	S_TCC CCC AAC TTG AGA TGT ATG AAG	Actin, cytoplasmic 1	P60709
		AS_AAC TGG TCT CAA GTC AGT GTA CAG G		

*5′ACTB*	NP001092	S_ATC CCC CAA AGT TCA CAA TG	Actin, cytoplasmic 1	P60709
		AS_GTG GCT TTT AGG ATG GCA AG		

S: sense, forward primer 5′ to 3′; AS: antisense, backword primer 3′ to 5′.
